# Challenges and opportunities for telehealth in the management of chronic obstructive pulmonary disease: a qualitative case study in Greece

**DOI:** 10.1186/s12911-020-01221-y

**Published:** 2020-09-10

**Authors:** Violeta Gaveikaite, Casandra Grundstrom, Stefan Winter, Helen Schonenberg, Minna Isomursu, Ioanna Chouvarda, Nicos Maglaveras

**Affiliations:** 1 Laboratory of Computer Science, Medical Informatics and Biomedical Imaging Technologies, School of Medicine, Faculty of Health Sciences, Aristotle University of Thessaloniki, 54124 Thessaloniki, Greece; 2 Department of Collaborative Care Solutions, Philips Research, High Tech Campus 34, 5656AE Eindhoven, The Netherlands; 3 M3S, Faculty of Information Technology and Electrical Engineering, University of Oulu, Pentii Kaiteran katu 1, 8000, FI-90014 Oulu, Finland; 4 Department of Collaborative Care Solutions, Philips Research, Pauwelsstraße, 17 52074 Aachen, Germany; 5 Department of IEMS,McCormick School of Engineering, Northwestern University, Evanston, IL USA

**Keywords:** Telehealth, COPD, Chronic obstructive pulmonary disease, Integrated care, Case study, Opportunities, Challenges

## Abstract

**Background:**

Telehealth (TH) was introduced as a promising tool to support integrated care for the management of chronic obstructive pulmonary disease (COPD). It aims at improving self-management and providing remote support for continuous disease management. However, it is often not clear how TH-supported services fit into existing pathways for COPD management. The objective of this study is to uncover where TH can successfully contribute to providing care for COPD patients exemplified in a Greek care pathway. The secondary objective is to identify what conditions need to be considered for successful implementation of TH services.

**Methods:**

Building on a single case study, we used a two-phase approach to identify areas in a Greek COPD care pathway where care services that are recommended in clinical guidelines are currently not implemented (challenges) and areas that are not explicitly recommended in the guidelines but that would benefit from TH services (opportunities). In phase I, we used the care delivery value chain framework to identify the divergence between the clinical guidelines and the actual practice captured by a survey with COPD healthcare professionals. In phase II, we conducted in-depth interviews with the same healthcare professionals based on the discovered divergences. The responses were analyzed with respect to identified opportunities for TH and care pathway challenges.

**Results:**

Our results reveal insights in two areas. First, several areas with challenges were identified: patient education, self-management, medication adherence, physical activity, and comorbidity management. TH opportunities were perceived as offering better bi-directional communication and a tool for reassuring patients. Second, considering the identified challenges and opportunities together with other case context details a set of conditions was extracted that should be fulfilled to implement TH successfully.

**Conclusions:**

The results of this case study provide detailed insights into a care pathway for COPD in Greece. Addressing the identified challenges and opportunities in this pathway is crucial for adopting and implementing service innovations. Therefore, this study contributes to a better understanding of requirements for the successful implementation of integrated TH services in the field of COPD management. Consequently, it may encourage healthcare professionals to implement TH-supported services as part of routine COPD management.

## Background

COPD is a chronic disease associated with an increased incidence of morbidity and mortality worldwide [1]. COPD-related exacerbations, which often result in hospital re-admissions [2], have a substantial impact on the quality of life of patients as well as on healthcare resources [3]. To illustrate this, the COPD-related hospital admission rate in 2009 in the European Union was 184 per 100,000 persons a year, exceeding asthma-related admissions almost threefold [4]. To minimize the societal burden and improve patient outcomes, more effective disease management is necessary.

The World Health Organization describes effective COPD management as a prevention of disease progression, symptoms relief, improved exercise tolerance and enhanced health status, prevention and treatment of complications and exacerbations, and reduced mortality [5]. In other words, the goals of chronic disease management are not related to a cure, but rather to minimizing symptoms through secondary prevention and to enhancing both functional status and quality of life [6]. Traditional healthcare approaches that focus on a specific disease are failing to meet the requirements of effective management [7] because COPD patients are often multimorbid [8]. In this light, embracing a more patient-centered approach that includes patients and healthcare professionals (HCPs) working together to optimize disease management is favorable [7]. Therefore, improvements in healthcare processes and advances in technology, such as those that allow for the implementation of telehealth (TH), are necessary developments [9].

TH is the ongoing and remote exchange of data between patients at home and healthcare professionals as part of disease management [10]. TH is expected to enable patient-centered care and offer solutions to tackle the problems of chronic disease management [7]. While early studies show that TH services reduce the utilization of healthcare services and the workload of HCPs, this is not a universally accepted truth [11–13]. In addition, TH is expected to promote and enable patient self-management [14]. Patient self-management is vital in chronic diseases management [15], but very challenging to implement in actual clinical care because of the organizational issues or education skill deficit [16]. Therefore, a strategy on how to promote and support patient self-management in actual clinical care should be developed and subsequently advocated.

One reason for the debatable impact of TH is the episodic care structure in healthcare systems. When considering care more continuously, integrated care (IC) and value-based care (VBC) are paradigms that try to tackle this problem through process innovations [17]. IC focuses on process innovations and value-case development [18]. This allows the advancement of a shared vision and setting common goals across different providers or teams. A value case looks beyond the potential financial returns of an individual stakeholder towards the benefits for patients and the community as a whole [18]. In VBC theory, value is defined as the health outcomes that matter to a patient over a complete cycle of care, per dollar spent [19]. A clear advantage of VBC is availability of strategic toolset for implementation in actual clinical care [17]. For example, adequately named care delivery value chain (CDVC) offers a systematic framework to delineate and analyze the process of care delivery for a specific medical condition. This framework helps to understand the diverse aspects of care delivery, how disease relates to other care processes, and where structural improvements can be made [19].

One strategy to improve care delivery is the creation or improvement of a care pathway. Care pathways are structured multidisciplinary plans which coordinate essential steps in the care of patients with a specific clinical problem [20]. There have been several attempts in Europe to build COPD care pathways. However, they currently do not incorporate TH services at scale [21]. This may be explained by the limited evidence for positive outcomes related to TH services implementation of TH services into the actual clinical practice presented in current clinical practice guidelines [2].

A recent COPD care pathway mapping effort showed five European macro care pathways [22]. Interestingly, the five care pathways were quite different when considering them from the perspective of patient referral systems, the maturity of technological infrastructure, and stakeholder involvement [22]. Country-specific challenges require more innovative approaches, such as TH, to improve IC and enable patient self-management. This study will contribute to the research stream by investigating one such care pathway in more detail with a focus on pinpointing the potential advantages of TH services. This paper addresses the following research question: What opportunities for TH and challenges are perceived by HCPs in a COPD care pathway?

To address this research question, we use Greece as a country-specific context to focus on a specific COPD care pathway as a case study. We explore the perspectives of four HCPs by combining different qualitative data collection methods to gain better understanding of what conditions need to be facilitated to successfully implement TH.

## Methods

### Definitions

A challenge is defined as a care service that is recommended in clinical guidelines but missing in current clinical practice. An opportunity is defined as a TH care service that is not present in current clinical guidelines or practice but is expected to bring benefits or value to relevant stakeholders. An innovation is defined as a TH care service that either contributes to solving a challenge or introduces an opportunity.

### Case study

**Fig. 1:**
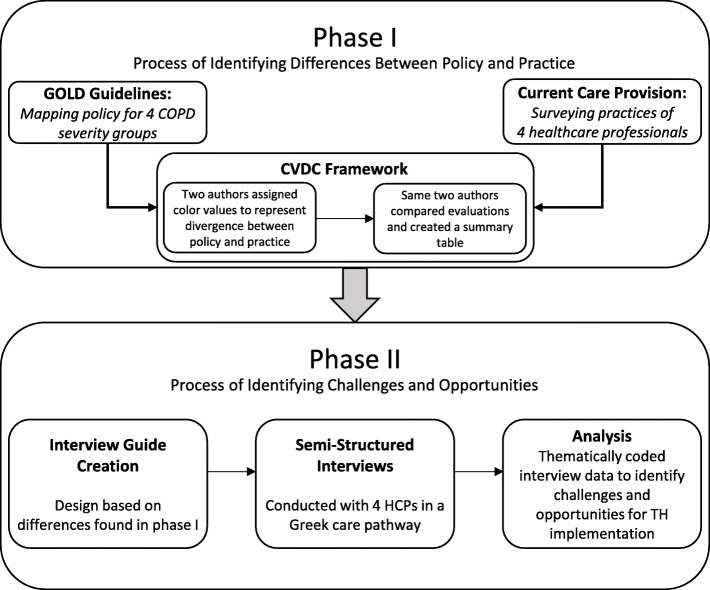
Study methodology

The study methodology is depicted in Fig. 1. For the purposes of this study, the instrumental case study approach was selected as it is appropriate to generate an in-depth appreciation for a particular situation [23]. The flexibility of a case study approach is uniquely suited for the goals of this project, as it offers a natural understanding of the complex phenomena of practices and perceptions in a care pathway [24]. This case study was performed in the Asclepius hospital (Thessaloniki, Greece)^1^, which is a referral center for respiratory diseases in Greece. Four different HCPs have been surveyed and interviewed (Table 1). More detailed participant characteristics including job description and tasks have been published previously [25]. The governance structure of the Asclepios hospital could be defined as hierarchical, where top-down leadership is present in all levels of the organization. The director is appointed by the health minister and supported by a management board that includes medical doctors. The diagnosis-related group system is not yet present. However, it is under active central development and will be tested soon. The budget is fixed and defined by the management board. There are frequent budget cuts and poor flexibility due to the ongoing economic crisis in Greece. In lower management levels, self-governance is present at the department level. Quality controls are implemented through a top-down approach and governed by doctors. There is a lack of performance-based evaluation, which means that there are no annual goals or targets set at any level in the organization.

**Table 1:** Participant characteristics in Greece care pathway

*Participant ID*	*Job Title*	*Job Location*
1	Head Nurse	Public healthcare hospital
2	Physiotherapist	Public healthcare hospital
3	Pulmonologist (in training)	Public healthcare hospital
4	Pulmonologist	Private practice with referrals to public healthcare hospital; the gate keeper

Participant ID number corresponds to answers in the results section

The hospital is organized in three parts for treating respiratory conditions, including COPD: in-hospital unit, emergency department (ED) and outpatient clinic. It was not possible to retrieve the number of COPD-related visits to the hospital. There were about 5000 respiratory-related outpatient clinic visits in 2016, approximately 2400 ED admissions and 2100 patients were hospitalized. Mean hospital admission duration was 4.2 days. The in-hospital respiratory department consists of 6 doctors, 12 permanent nurses, several nursing staff assistants, one physiotherapist and one spirometry specialist. The department is an active regional participant in scientific research and actively involved in providing training programs for a variety of clinical trainees/residents.

The participant selection process was based on the COPD care pathway reported in relevant literature in Greece; each participant represents one of four major HCPs in the care pathway [22]. In Greece, the pulmonologists have a “gatekeeper role” for stable COPD patient management which means, that in case of any problems patients first consult the pulmonologist, who in turn can refer them to other specialists such as pulmonary rehabilitation specialists. Patients with exacerbations are treated in the hospital, followed by inpatient or outpatient care during follow-up visits [22].

The four HCPs are the primary stakeholders involved in the care of patients with COPD in Greece. They are often participating in research projects which are designed to deliver remote care [22]. Professional help with social or psychological issues is not a common practice in Greece, as family members traditionally play a significant role in patient care. However, psychiatric care is part of comorbidity management in this care pathway. In the Asclepius hospital, the average COPD caseload is around 15–20 patients per week. The hospital has a catchment area that includes the urban areas of Thessaloniki as well as more remote, rural areas. The public healthcare system is the focus of the case study. Convenience sampling was used to recruit HCPs [26]. All healthcare professionals are actively participating in the care pathway for COPD patients and permanently employed at the Asclepius hospital.

### Data collection and analysis

Data collection and analysis this case study was a sequential process of two iterative phases with an increasing level of detail, where data was both collected and analyzed in each phase.

### Phase I – identification of divergences between policy and practice to inform the creation of an interview guide

The goal of this phase was to prepare for the creation of the interview guide. First, the GOLD guidelines were mapped into the CDVC framework (policy CDVC). Second, HCPs filled in the CDVC framework based on their actual practice (practice CDVC). Third, the divergence between policy CDVC and practice CDVC was used to inform the interview guide creation process.

National COPD guidelines are used to evaluate the policy context for this case study which took place during March 2018. We adopted the CDVC framework as a mapping tool to understand the divergence between the clinical guidelines and existing evidence-based care practices [19]. The mapping of the guidelines allowed for a structured and systematic approach to understanding the different care processes for each COPD severity. The use of surveys is a common tool accepted by HCPs as a flexible and non-intrusive method of gathering data [27]. The survey retained the original CDVC structure which corresponds to six areas of our interest. These areas are as follows: monitoring and prevention, diagnosis, intervention preparation, intervention, recovery and rehabilitation, and monitoring and management. The survey asked which clinical guidelines were followed by the participants, if any, as well as provided examples for COPD care considerations for informative purposes. The survey was color-coded to allow for ease of reading and structured filling out. The survey template is available in Additional file 1. Surveys were distributed to the HCPs during March 2018. The completed surveys were collected in June 2018. Four HCPs from along the COPD care pathway in Asclepius hospital (see Table 1) filled them out individually. To further allow for triangulation – the mixing of data types from multiple sources to facilitate validation [28] – all additional documentation provided by the clinical participants were included in the analysis process. The divergence between policy and practice was determined by matching the guidelines (Additional file 2) to the practices of the healthcare professionals. To perform this task, two authors (VG and CG) individually assigned color values to represent the completeness of the practices against policy for each HCP. Then the same two authors compared their individual evaluations and created a summary table, which depicted the level of which healthcare provision was compliant with policy (Additional file 3). To depict compliance, stop-light color coding was used. Observed in Phase I were used to focus the semi-structured interviews in Phase II.

### Phase II - interview guide creation for challenges and opportunities

The goal of this phase was to explore the challenges and opportunities for the implementation of TH-supported services in the Greek care pathway. First, a semi-structured interview guide was designed. Second, interviews were conducted with the same four healthcare professionals who were surveyed in Phase I. Third, interviews were thematically analyzed for challenges and opportunities of TH implementation.

The semi-structured interview guide was designed based on the divergence between policy and practice in Phase I. The four areas which showed a significant discrepancy between policy and practice were: diagnosis, intervention preparation, intervention, and recovery and rehabilitation. The semi-structured interview guide is available in Additional file 4. No specific TH interventions were mentioned during the interview to prevent influencing the HCPs’ perception of TH.

The semi-structured interviews were conducted at pre-arranged and mutually convenient times and lasted between 30 and 60 min. The interviews closely followed the interview guide to ensure homogenous data collection. All interviews were transcribed following a denaturalization process to remove language idiosyncrasies while also remaining faithful to meanings and perceptions of the interviewee [29]. The framework method from Gale et al. [30] was followed to ensure an organized and systematic coding process, leading to the generation of meaningful themes. The theming process and analysis was performed without specialized software. The framework method was chosen as it is an appropriate management tool for multi-disciplinary research teams in health service research, and provides structure during the analysis of qualitative data [30]; important for our study due the varying levels of qualitative experience.

## Results

The study type is considered to be instrumental, meaning *Phase I* results are used towards informing the main results section in *Phase II*.

### Phase I – groundwork for the interview guide creation

In Asclepius, the main COPD care document is the GOLD guidelines [31], which is used by all HCPs. Patients are classified based on the impact of COPD on the patient using the recently introduced ABCD assessment tool. This tool combines the assessment of symptoms with the patient’s spirometry classification and/or risk of exacerbation [31]. The guidelines indicating COPD policy are available in Additional file 2.

The differences in clinical care processes between clinical practice and clinical guidelines are available in Additional file 3. The two areas proven to match well between policy and practice were: monitoring and prevention before COPD diagnosis, and monitoring and management after the patient is discharged. Three areas, namely diagnosis, intervention, and recovery and rehabilitation, only partially matched between policy and practice. The cross-section representing interventions for very severe patients, was found dissimilar to policy guidelines. Finally, clinical participants did not mention the preparation of interventions.

### Phase II – perceived challenges and opportunities

#### Challenges within self-management and comorbidity management

In this section, we present the challenges within five key areas: patient education, medication adherence, physical activity, smoking cessation, and comorbidity management. Self-management includes patient education, medication adherence, physical activity and smoking cessation.

##### Patient education

In Greece, both the pulmonologist working in the hospital and the private practice specialist teach dyspnea management and coughing techniques during patient visits or distribute take- home materials. In Greece, a hot and humid country, weather-based events are significant environmental factors for HCPs to consider. Both medical doctors highlighted that they advised patients during visits on climate-related issues: *“It’s very difficult for them. During the summer, the temperature is very high … we instruct them to go outside or to do their shopping early in the morning or late at evening. And here especially in Thessaloniki, we have a lot of humidity, which is something that might impact the respiratory system” (4 - Pulmonologist).* Awareness about issues in winter are also stressed: *“I give the instructions during the winter [regarding] the fireplaces…” (4 - Pulmonologist).*

The interviews suggest that the role of clinicians in patient non-pharmacological management was to provide the necessary information and clear recommendations to patients. The pulmonologist expressed concerns about the efficacy of paper flyers and whether or not patients actively participate in self-education at home. *“Most of [the patients] do not even read [the flyers]. We are asking and we are trying to see if they are doing everything we discussed in the previous visit” (4 - Pulmonologist).* Considering both roles, a healthcare provider distributing take-home materials in the form of flyers indicates that only verbal and passive patient education takes place. The majority of patients do not show self-sustaining home-behaviors for self-education.

A problem discussed by the HCPs is the relative lack of doctors considering the number of patients. This means HCPss must prioritize topics to discuss with the patient, which may not always include support for self-education: *“It’s difficult, we have too many patients and we are very few doctors... We don’t have ability to tell them many things” (3 - Pulmonologist).* The challenge within education is found in conveying impactful education on a large scale that is meaningful for COPD patients.

##### Medication adherence

Doctors are responsible for informing the patient on correct medication usage (informing self-management), including inhalation techniques. However, adherence to medication and proper use outside of the hospital is the responsibility of the patient (sustaining self-management). Our interviews revealed that the emphasis on medication adherence support is adopted by clinicians to ‘motivate’ patients. The practice of informing about self-management for medication adherence requires doctors to allocate specific time during the patient visit. A nebulizer is a drug delivery device that requires inhaler technique training provided during the patient visit. As the inhaler technique training is extensive and demands specific skills, the time it takes to inform about, train, and practice the correct technique is quite high. *“When I start to give an inhalation device to my patients, first thing I do I explain how it works and I have a device for demonstration they can [practice on]” (4 - Pulmonologist).*

However, ensuring that the tool is being used correctly at home, relies on the patient’s understanding during the patient visit. *“I ask to show me how they take the medication. If they don’t take them properly, I have a device for demonstration to show them again and again how they can take medicines” (3 - Pulmonologist).* This illustrates that medication adherence at home is at least partially dependent on doctors informing patients about it during the visit.

Administering the correct dosage of medication when the patient is at home is also important for COPD self-management. The doctors rely upon patient recollection through feedback prompting to paint a picture of adherence. *“I ask them how many times they take a medication a day...” (3 - Pulmonologist).* Forcing the doctors to trust what the patient is reporting and relying on the accuracy of subjective evaluation. *“You have to believe what they saying to you, but if the patient doesn’t do exactly what are you [advising]” (4 - Pulmonologist).* However, both doctors reported that they might be suspicious of poor medication adherence for a variety of reasons. For example, the correct usage of an inhalation device if the patient is showing symptom deterioration. To support medication adherence at home, no technology tool is available. The challenge within medication adherence is how to both inform on and sustain patient self-management.

##### Physical activity

The physiotherapist by training, and pulmonary rehabilitation specialist by title has the primary responsibility for physical activity management of the care pathway in Asclepius hospital and provides an unstructured rehabilitation program. Asclepius adheres to the practice of directing patients to pulmonary rehabilitation specialists through a set process. Both medical doctors reported that a physiotherapist should provide information on physical activity, as care beyond the walls of the hospital is not part of the job scope: *“Here in Greece we don’t have [physical activity training] … we refer them to physiotherapist” (4 - Pulmonologist).*Physical activity programs do exist in some Greek hospitals, but the doctors interviewed do not know anything about it: *“There is such a program in another hospital, but I don’t participate, so I can’t tell you about that” (3 - Pulmonologist).*

The physiotherapist expressed that although physical activity is a crucial part of managing stable COPD, other priorities take place in their work practices such as informing on cough, dyspnea, and sputum production. *“Physical activity is very important but when [the] patient comes to the hospital the first [focus] is the cough, not a physical activity, this is after” (2 – Physiotherapist).* Part of the reason why sustaining remote physical activity practices are not in place is because the physiotherapist cannot visit the home of the patient. Instead, the burden of choice for physical activity is placed squarely on the shoulders of the patient. *“[The patient] can decide if he wants to go to[a] private clinic or to [a] place to continue [the physical activity]” (2 – Physiotherapist).* The physiotherapist informs on using a static bike at home either in bed or in a chair when the patient is hospitalized.

Apart from not being able to visit the patient at home, the physiotherapist also cannot prioritize providing physical activity consultations due to more critical self-management elements of which they are responsible *“... the cough and the dyspnea [are] the most important” (2 – Physiotherapist).* The challenge within physical activity is providing physical activity to COPD patients in either the rehabilitation or home setting.

##### Smoking cessation

The interviews revealed that in the public hospital no information on, or follow-up for smoking cessation is provided. In Greece, smoking cessation intervention is the responsibility of smoking cessation clinics and pulmonologists trained to provide smoking cessation services. In the care pathways of Asclepius, smoking cessation specialists are not part of care. *“We don’t have specialized doctors. And we advise them to visit [a] specialized doctor and this topic” (3 - Pulmonologist).*

Even though the pulmonologist is specialized in smoking cessation, these services are only performed in a separate, private clinic specializing in pulmonary rehabilitation. The pulmonologist mentioned and actively supports smoking cessation for COPD self-management, but the doctors have limitations. *“If you want to manage smoking cessation, you need to be specialized as [a] pulmonologist. If you are not specialized pulmonologist in the smoking cessation you … have to tell the patients [to] go [to the smoking clinics] for smoking cessation …” (4 - Pulmonologist).*

The nurse and physiotherapist have no role in supporting or making decisions on smoking cessation. Additionally, the pulmonologist receives limited feedback when a smoking intervention is carried out. Instead, all responsibility for smoking cessation shifts to the patient as no informing or sustaining activities are taking place during patient visits or hospitalization events. The challenge within smoking cessation is facilitating access to smoking cessation programs, and follow-up after the program takes place.

##### Comorbidity management

The main three comorbidities that were found to significantly impact COPD management were: diabetes, heart failure, and depression. The nurse and the physiotherapist are not part of managing, treating, or making decisions related to comorbidities. *“[We] wait for a doctor to give instructions, what to do with medications and the care with a patient”* (1 – Head Nurse).

When dealing with comorbidities, the doctors were shown to be meticulous to check patient comorbidities *“I ask them every time about their medical history, even if I know medical history, I ask them again, and again every follow up. I want them to show all medications they take, how many times a day” (3 - Pulmonologist).* Some collaboration efforts are in place to ensure comprehensive patient treatment *“we work together with a specialist. For example, if I have a COPD patient with heart failure, I would talk with cardiologist so to manage therapy protocol together for the heart failure and for COPD.” (4 - Pulmonologist).*

The participants indicated a lack of electronic records (EHR) means that most work is still done manually and in paper format. The challenge within comorbidity management is administration for tracking and managing a complex chronic disease with additional HCPs.

#### Opportunities to support patients

##### Telehealth as a bi-directional communication tool

Participants mentioned that TH would facilitate more efficient access to patients: *“It will be easier for the doctor to access the patient, and it would be easier for the patient to access the doctor” (4-Pulmonologist).* The most important factor to highlight here is that access takes place in a bi-directional setting; the patient should have access to the doctor, and the doctor should have access to the patient. At the moment, the communication is initiated by the patient and usually triggered by a disease-related event.

The pulmonologists emphasized a potential shift towards patients taking more responsibility for their health because of TH intervention support and the capability to contact HCPs in case of a significant worsening of condition: *“I want him to call me when he has very severe symptoms” (3-Pulmonologist).* The TH system should also automatically notify HCPs when the clinical condition of a patient deteriorates: *“Or the system to call me when these symptoms [change]…” (3-Pulmonologist).*

The pulmonologists expressed a desire to stay in control of final decision making, meaning a TH system should support the capability of a decision maker but be limited to triage: *“I don’t want the system to decide what is more important for a patient” (4- Pulmonologist*).

TH was also seen as a tool for HCPs to reduce the time intervals between appointments with patients: *“[I] don’t need to wait for an appointment with a patient to see if he has a problem. With telco, [I] can directly give advice [to patients]” (2- Physiotherapist).* When considering remote areas where healthcare resources are scarce, having access to TH services would facilitate regular patient-doctor contact: *“Especially here in Greece, we have areas that are very far away, and they don’t have doctors and they don’t have nurses and so the TH will be able to stabilize it” (1* – *Head nurse).* Healthcare service delivery and experiences for all stakeholders could be improved by TH.

##### Telehealth as a tool to reassure patients

Asclepius mainly manages severe cases of COPD, and many COPD patients have a sensitive psychological state, with depression being one of the most frequent comorbidities. The unexpected nature of exacerbations, more common in severe cases of COPD, keep patients in a constant state of anxiety: *“Don’t forget that these patients with stage three or four, they are very anxious about their health, if they call to the doctor, [the] doctor needs to immediately answer they call” (3* – *Pulmonologist).*Participants shared considerations that TH would help reduce patient anxiety because HCPs can monitor and respond to a patient’s status: *“So if you have telehealth system for these patients it would be better. Because they would know that the doctor has access to their data all the time. [The patients] know that the doctor will call them, or they can call the doctor to manage their problem” (3-Pulmonologist)*. It was further emphasized by all participants that TH interventions would increase patient-safety as well.

## Discussion

### Challenges and opportunities in providing care for COPD patients

There is limited qualitative research available, which details care pathway challenges and opportunities to empirically inform about possible care service solutions, such as TH. Our study contributes to this research area by illuminating the divergence between clinical guidelines and actual clinical care. There are four particular areas of clinical guidelines which should be emphasized for future TH implementation in the Greek care pathway. These are: diagnosis, intervention preparation, intervention, and recovery/rehabilitation. Self-management components for care provision are the most challenging and require a different organizational approach to be implemented and enforced in the actual care pathway. However, when comparing current literature with our findings, there is a striking difference as most studies focus on pharmacological management or medication adherence [32, 33]. Our research was focused on a non-pharmacological management for COPD. For instance, a study in Greece reported a low adherence to guidelines regarding treatment and highlighted several reasons for non-adherence [33]. It suggests that non-pharmacological management was not considered to be a care element.

Current practices may harbor prospects for TH implementation through the five challenges identified within self-management and comorbidity management for the COPD care provision. These were: education (conveying effective and impactful education on a large and meaningful scale), medication adherence (informing and sustaining patient medication behaviors), physical activity (facilitating rehabilitation in outpatient settings), smoking cessation (providing support for smoking cessation programs), and comorbidity management (managing administrative duties for a complex network of HCPs). All these elements are discussed in the GOLD guidelines [2]. It is important to emphasize, that these elements are discussed as individual elements. It is not yet clear how they can be combined. Some systematic reviews address that a self-management plan and its technical support might be beneficial for COPD patients. However, the evidence is not yet conclusive [34, 35] We suggest there is a need for research to determine not only the efficacy of TH for COPD management but also to utilize qualitative research to aid in the understanding of adoption and sustainability mechanisms when considering pathways and their features.

Difficulties in educating patients were seen as a communication challenge by clinical stakeholders due to a lack of effective and large-scale means to reach patients. This indicates a prospect for TH implementation because it allows remote patient education in large groups, does not require time scheduling and allows HCPs to sustain patient interest in self-management through education. A recent systematic review supports this finding, showing that education of chronic patients delivered through virtual modalities was comparable, or more effective than standard care [36]. This is important, since it demonstrates effectiveness of virtual education for chronic patients. The next step would be a large-scale implementation.

HCPs reported that informing and sustaining patient medication behaviors was a challenging task because the patient has a disease with complex medication needs. TH could support medication adherence because it offers a chance to track patients remotely. For instance, some trials incorporated the medication adherence report scale in their trial [37] or their daily symptom diary [38]. However, systematic knowledge towards managing medication adherence for COPD with TH tools is not present. This could be in large part due to few clinical trials using TH that report on medication adherence. Since there is a significant lack of evidence to support medication adherence with TH, future trials investigating TH should include medication adherence as an outcome.

Facilitating physical activity for COPD is significant part of rehabilitation because it is a strong predictor of COPD patient mortality [39]. Our physical therapist considered TH to be useful in the outpatient setting as it improves their access to patients and increases patient activation. Considering physical activity support by TH, Lundell et al. stated that TH may increase patients physical activity levels [40]. Moreover, recent studies show the potential of TH to increase or maintain physical activity when managing COPD patients [41, 42].

In COPD, smoking cessation is one of the most critical steps to positively influence the disease course [2]. In the Greek care pathway, the challenge within smoking cessation is restricted as HCPS have limited ability to provide smoking cessation programs. This finding is in line with report released in 2011, which stated that only 3% smokers ever visited a smoking cessation office [43].

In addition, if the patient was referred to the smoking cessation clinic, his “treatment” outcome and follow-up was fragmented and did not reach the COPD care gatekeeper. TH would be a candidate to support such a service, as it provides remote access, follow-up possibilities and timely communication to boost patient motivation. In addition, sufficient clinical evidence is available to support the use of web -based smoking cessation programs for adult smokers in clinical practice [44]. Before implementing this solution in TH services, contextual factors should be considered.

Implementing web- or mobile application-based TH solution here could be an option, as it would provide timely access to the data for all stakeholders and facilitate decision making towards medication prescription or the COPD-exacerbation related diagnosis. However, there is little evidence to conclude that comorbidity monitoring is beneficial. Despite the lack of evidence, Bourbeau et al. propose to explore the area of the integrated remote monitoring of COPD-related comorbidities [12].

Bi-directional communication and TH as a tool to reassure patients were reported by our participants as opportunities for TH services. Our findings were consistent with opportunities reported in other studies. For instance, Ure et al. [45] discussed the importance of timely communication to prevent adverse disease outcomes, although bi-directional communication was not emphasized. The communication advantages were reported from different perspectives where patients felt happy with the chance to communicate with their HCP’s. However, some nurses felt that TH limits their ability to understand the patient’s condition and needs [46]. The nurse in our case study contradicts this attitude, and instead sees TH as a tool to facilitate better and different patient relationships [25]. Regarding our case, Greece has a lot of remote territories (up to 25%) and TH could be used as a service to secure patient-HCP communication, especially when considering medical staff shortages and economic crisis [47]. Huniche et al. [48] discussed “a sense of security” as a positive patient experience. However, it might be a misleading one, as it transfers responsibility away from HCPs, and towards the TH service.

## Conditions for implementing Telehealth

Before implementing TH in routine care, multiple dimensions such as structure, process, and patient outcomes should be considered [49, 50]. Structure describes the stable elements of care organizations (for example, facilities, education or technology). Within this dimension, four elements are worth mentioning in the Greek care pathway: the impact of the current economic crisis, IT infrastructure, the gatekeeper role for COPD care, and specialized nurse integration in the care organizational model.

The healthcare IT environment in Greece is limited for a variety of reasons. EHRs are not fully integrated into the system [51], which results in difficulties with care coordination and timely information transfer [52]. There is no policy to encourage the adoption and maintenance of high quality EHRs. The current economic crisis in Greece may shift healthcare priorities to other areas of interest [53]. In addition, more than 40% of doctors report emotional exhaustion or depersonalization, which might impact their willingness to adopt innovations [53]. Therefore, the climate for innovations when considering new healthcare services, require additional funding which might be turbulent [54].

In Greece, the role of gatekeeper connecting patients to the various service providers belongs to pulmonologists. This is a major concern, as they are heavily overburdened by work [22]. In most European countries, the gatekeeper role belongs to primary care providers [22]. Primary care in Greece is mainly responsible for identifying new cases. However, more than 50% of new COPD patients are not correctly diagnosed, resulting in over-diagnosed cases culminating in unnecessary medication costs [55]. We consider the economic crisis to have a silver lining for the fortuitous timing to advocate for changes that would be beneficial to a variety of healthcare stakeholders [56]. Potential care provision benefits could include allowing integration of specialized nurses into clinical teams or implementing TH for COPD self-management. While nurses can obtain specialized training in Greece, they are not allowed by law to perform the more specialized tasks they are trained to perform. Considering that TH implementation necessitates the introduction of new roles by nurses, the law limitation for nurses in Greece may endanger TH sustainability. Our interviews indicate a certain level of readiness for change from the HCPs as they see many problems in the current environment and consider TH as a tool to help manage them.

Interestingly, in Greece, local healthcare providers are actively participating in TH efficacy trials for COPD management [57, 58]. Indicating that HCPs would already have well-formed opinion towards implementing TH. Considering TH implementation, the right profile of stakeholders is important. Patients should be willing and able (for example, concerns about patient IT literacy) to use technologies, and HCPs should be educated to use TH and allocated patient data revision as part of care practice.

## Strengths and limitations

This study represents a single case study from a COPD care pathway in Greece, and has certain limitations. First, the use of the CDVC framework has not been applied for COPD disease management in the literature previously. Although several justifications were made for suitability, there is some risk to applying methodological approaches in new contexts. Ideally, testing the CDVC framework for mapping clinical guideline accuracy would have taken place as an individual research method protocol.

Second, the sample size of four HCPs may be considered small. However, each role in the COPD care pathway of Asclepius was represented, providing rich data material. The gathered information is limited by the lack of ethnographic details in the current study, impacting the external validity of the study [59].

Third, the authenticity of this study was validated independently by the four interviewed HCPs. Even though some English language fluency was a prerequisite for the semi-structured interview, some terminology was difficult to understand for some stakeholders. Due to this, the chief pulmonologist participated in the discussions and helped to facilitate the interviews. This participation may have led to a discrepancy between a given answer in Greek and its translation.

Fourth, some of HCPs have participated in TH pilot studies which may have influenced their perceptions of TH. However, we consider this experience with TH to be beneficial for our research as they had a wider variety of opinions.

## Conclusions

Although the opportunities and challenges of delivering care to COPD patients are similar across Europe, the solutions to tackle these challenges may require very different approaches. Future research should aim to develop an in-depth understanding of the opportunities for TH and challenges of TH implementation in care pathways through longitudinal and complementary data collection methods, such as ethnography. For optimal results, the attitudes and perceptions of HCPs who use these TH tools should be considered as they provide in-depth and practical insights. Moreover, additional stakeholders, such as patients and their relatives, should participate in the research activities to provide a more holistic picture. In the country-specific setting of Greece, tailored TH solutions should be implemented in care pathways to support HCPs and patients with the management of comorbidities and self-management.

## Supplementary information

**Additional file 1.** Visualized Representation of CDVC tool for the care practice mapping. Each disease severity category had a different tool, in total four tools, distributed for 4 clinical stakeholders. Each stakeholder should report which procedure they provide to the patient in the table’s cross-section.

**Additional file 2.** Visualized GOLD Guideline Mapping Summary. IC = Integrated Care; TH = Telehealth; *Special Conditional Notes if patient is Exacerbating 1 Resting chronic hypoxemia for Long-term oxygen therapy 2 Non-invasive ventilation 3 Continuous positive airway pressure 4 Chronic Care Model 5 https://www.ncbi.nlm.nih.gov/pubmed/24127811 6 https://www.ncbi.nlm.nih.gov/pubmed/2713226

**Additional file 3.** Visualized Representation of Policy and Practice. ABCD are the GOLD COPD disease classification categories. White represents exact or similar practices to policy; blue, some absent practices to policy; orange pattern, no similarity from practice to policy; white with crosses, no responses.

**Additional file 4.** Semi-Structured Interview Guide.

## Data Availability

The datasets generated during and/or analyzed during the current study are not publicly available to prevent disclosure of personal information.

## Footnotes

1 For ease of referencing, we will refer to the case study site as ‘Asclepius’ for anonymization purposes, and to prevent revealing the identity of the hospital and/or staff.
