# Supplementary material for: Challenges and opportunities for telehealth in the management of chronic obstructive pulmonary disease: a qualitative case study in Greece
Source: BMC Med Inform Decis Mak. 2020 Sep 10;20:216. doi: 10.1186/s12911-020-01221-y (PMC7488260; doi:10.1186/s12911-020-01221-y)
Supplement: Supplementary file 1 — Additional file 1. Visualized Representation of CDVC tool for the care practice mapping. Each disease severity category had a different tool, in total four tools, distributed for 4 clinical stakeholders. Each stakeholder should report which procedure they provide to the patient in the table’s cross-section. [file 12911_2020_1221_MOESM1_ESM.docx]

Care delivery value chain (CDVC) - **GOLD I** (or other disease severity classification system)

|  | **Monitoring / prevention**  **(MP)** | **Diagnosis**  **(D)** | **Intervention preparation**  **(IP)** | **Intervention**  **(I)** | **Recovery / rehabilitation**  **(RR)** | **Monitoring / management**  **(MM)** |
| --- | --- | --- | --- | --- | --- | --- |
| **1. List of the procedures for COPD patient** | List **MP** procedures before disease diagnosis  *Ex: medical history review, risk factor review* | List **D** procedures used for disease management  *Ex: spirometry, lab testing, etc.* | List preparations for intervention procedures  *Ex:* counselling before surgery | List **I** procedures for disease management  *Ex: surgery, drug therapy* | List **RR** procedures  *Ex: Outpatient physical therapy, smoking cessation therapy* | List secondary prevention procedures  *Ex: adverse events monitoring, regular follow-up visits, etc.* |
| **2. Information** | How are patients informed about **MP** opportunities?  *Ex: national screening / Advice* | How are patients informed about **D** procedures? | How are patients informed about an intervention (prior)? | How are patients informed about **I** (after)?  *Ex: counselling on results* | How are patients informed about **RR**? | How are patients informed about secondary prevention?  *Ex: counselling on long term risks* |
| **3. Measurements** | Measurements taken at **MP** to monitor or prevent disease (primary prevention) | Measurements taken for **D** of disease | Measurements taken to prepare for an intervention | Measurements taken during **I** | Measurements taken in the **RR** phase | Measurements taken during follow up to monitor disease or for secondary prevention |
| **4. Access** | How do patients access screening facilities?  *Ex: national program, hospital visit, website, etc.* | How are patients referred for **D**?  Ex. *GP visit, ER admission* | How are patients referred for **IP**? | How are patients referred for **I**?  *Ex. GP visit, ER admission* | How do patients access **RR** facilities?  *Ex: outpatient clinic, physiotherapist, etc.* | How are patients monitored?  *Ex: laboratory testing, hospital visit, etc.* |

**Appendix 1-** Visualized Representation of CDVC tool for the care practice mapping. Each disease severity category had a different tool, in total four tools, distributed for 4 clinical stakeholders. Each stakeholder should report which procedure they provide to the patient in the table’s cross-section.
