# Supplementary material for: Challenges and opportunities for telehealth in the management of chronic obstructive pulmonary disease: a qualitative case study in Greece
Source: BMC Med Inform Decis Mak. 2020 Sep 10;20:216. doi: 10.1186/s12911-020-01221-y (PMC7488260; doi:10.1186/s12911-020-01221-y)
Supplement: Supplementary file 2 — Additional file 2. Visualized GOLD Guideline Mapping Summary. IC = Integrated Care; TH = Telehealth; *Special Conditional Notes if patient is Exacerbating 1 Resting chronic hypoxemia for Long-term oxygen therapy 2 Non-invasive ventilation 3 Continuous positive airway pressure 4 Chronic Care Model 5 https://www.ncbi.nlm.nih.gov/pubmed/24127811 6 https://www.ncbi.nlm.nih.gov/pubmed/2713226 [file 12911_2020_1221_MOESM2_ESM.docx]

| GOLD guidelines | **A** | **B** | **C** | **D** |
| --- | --- | --- | --- | --- |
| **Monitoring or Prevention** | History Factors of : Etiology, biology, occupational exposure, air pollution, genetic factors, age and gender, lung growth and development, socio-economic status,  and previous diseases | History Factors of: Etiology, biology, occupational exposure, air pollution, genetic factors, age and gender, lung growth and development, socio-economic status,  and previous diseases | History Factors of : Etiology, biology, occupational exposure, air pollution, genetic factors, age and gender, lung growth and development, socio-economic status,  and previous diseases | History Factors of : Etiology, biology, occupational exposure, air pollution, genetic factors, age and gender, lung growth and development, socio-economic status,  and previous diseases |
| **Diagnosis** | Same as Monitoring and Prevention A + airflow limitation, dyspnea, previous exacerbations, symptoms, and   comorbidities | Same as Monitoring and Prevention B + airflow limitation, dyspnea, previous exacerbations, symptoms, and   comorbidities | Same as Monitoring and Prevention C + airflow limitation, dyspnea, previous exacerbations, symptoms, and   comorbidities | Same as Monitoring and Prevention D + airflow limitation, dyspnea, previous exacerbations, symptoms, and   comorbidities |
| **Intervention Preparation** | Classifying patient based on ABCD tool, evaluating smoking status and physical capacity | Classifying patient based on ABCD tool, evaluating smoking status, physical capacity, and RCH1 | Classifying patient based on ABCD tool, evaluating smoking status, physical capacity, RCH1, and respiratory failures for NIV2 | Classifying patient based on ABCD tool, evaluating smoking status, physical capacity, RCH1,respiratory failures for NIV2, and CPAP3 |
| **Intervention** | Strategic selection of: smoking cessation, inhaler techniques, therapeutics, and deliver vaccination(s) | Strategic selection of: smoking cessation, inhaler techniques, therapeutics, and deliver vaccination(s). Evaluate: comorbidities impact | Strategic selection of: smoking cessation, inhaler techniques, therapeutics, and deliver vaccination(s). Evaluation of: comorbidities impact, nutritional status,  and possible evaluation of: hypercapnia treatment and NIV2 | Strategic selection of: smoking cessation, inhaler techniques, therapeutics, and deliver vaccination(s). Evaluation of: comorbidities impact, nutritional status,  and possible evaluation of: hypercapnia treatment, NIV2, palliative care, bronchoscopy and surgery |
| **Recovery and Rehabilitation** | X | Follow elements of: CCM4, education modules with personal feedback, and pulmonary rehabilitation5,6. Note: no evidence for IC or TH | Follow elements of: CCM4, education modules with personal feedback, and pulmonary rehabilitation5,6. Note: no evidence for IC or TH | Follow elements of: CCM4, education modules with personal feedback, and pulmonary rehabilitation5,6. Note: no evidence for IC or TH |
| **Monitoring and Management** | 1. Spirometry  2. Functional capacity  3. Blood gas sample  4. Symptoms evaluation  5. Exacerbations  6. Medication therapy  7. Comorbidities symptoms | Same as Monitoring and Management A +  8. Discussion on individual goals  9. Expiratory muscle and lower limb strength | Same as Monitoring and Management B | Same as Monitoring and Management B +  10. Follow-up for possible surgical complication |

**Appendix 2**- Visualized GOLD Guideline Mapping Summary. IC = Integrated Care; TH = Telehealth; *Special Conditional Notes if patient is Exacerbating 1 Resting chronic hypoxemia for Long-term oxygen therapy2 Non-invasive ventilation 3 Continuous positive airway pressure 4 Chronic Care Model

5 <https://www.ncbi.nlm.nih.gov/pubmed/24127811> 6 <https://www.ncbi.nlm.nih.gov/pubmed/2713226>
