# Supplementary material for: Challenges and opportunities for telehealth in the management of chronic obstructive pulmonary disease: a qualitative case study in Greece
Source: BMC Med Inform Decis Mak. 2020 Sep 10;20:216. doi: 10.1186/s12911-020-01221-y (PMC7488260; doi:10.1186/s12911-020-01221-y)
Supplement: Supplementary file 3 — Additional file 3. Visualized Representation of Policy and Practice. ABCD are the GOLD COPD disease classification categories. White represents exact or similar practices to policy; blue, some absent practices to policy; orange pattern, no similarity from practice to policy; white with crosses, no responses. [file 12911_2020_1221_MOESM3_ESM.docx]

| **Obstruction level by GOLD** | **A** | **B** | **C** | **D** |
| --- | --- | --- | --- | --- |
| Monitoring or Prevention before COPD Diagnosis |  |  |  |  |
| Diagnosis |  |  |  |  |
| Intervention Preparation | x | x | x | x |
| Intervention |  |  |  |  |
| Recovery and Rehabilitation |  |  |  |  |
| Monitoring and Management after Patient Discharge |  |  |  |  |

**Appendix 3 Visualized Representation of Policy and Practice**

ABCD are the GOLD COPD disease classification categories. White represents exact or similar practices to policy; blue, some absent practices to policy; orange pattern, no similarity from practice to policy; white with crosses, no responses.
