# Supplementary material for: Challenges and opportunities for telehealth in the management of chronic obstructive pulmonary disease: a qualitative case study in Greece
Source: BMC Med Inform Decis Mak. 2020 Sep 10;20:216. doi: 10.1186/s12911-020-01221-y (PMC7488260; doi:10.1186/s12911-020-01221-y)
Supplement: Supplementary file 4 — Additional file 4. Semi-Structured Interview Guide. [file 12911_2020_1221_MOESM4_ESM.docx]

Semi-Structured Interview Guide

Violeta Gaveikaite - Philips and Aristotle University of Thessaloniki

Casandra Grundstrom - University of Oulu

Telehealth Opportunities in Greek Clinical Practices for COPD

**Proposed Healthcare Professionals as Participants**

Nurse

Self-management specialist

Pulmonary rehabilitation specialist

Pulmonologist

**Areas of Interest While Designing Interview Questions**

Develop an understanding of the patient groups that are seen by healthcare professionals and correspondingly confirm severity levels and telehealth opportunities in order to be able to match with clinical guidelines

Discern the perceived burden of patient management and report on the frequencies of events and burden of practices where new services would be useful

To explore opportunities for addressing some of the healthcare professionals’ burdens through the use of telehealth services for COPD patients

1 Hour Proposed Time

Status: Draft

Reviewed by:

Ioanna Chouvarda – 19/07/18: changes made for clarity and content

Aleksandra Tesanovic – 20/07/18: changes made for flow, content, and neutrality

Ioanna Sokoreli – 20/07/18: changes made for prioritization and size

Jennifer Caffarel – 20/07/18: changes made for confirmation of suggestions from AT, and language choices

Pending:

Pilot test for length and clarity

Follow-up adjustments (minor)

The following are interview guide for use of the interviewer. When reading, please consider that the *italicized examples* after some questions is only to act as a reminder to the interviewer and not to be said aloud. The interview guide is divided into themed sections as indicated by the headers. Not all questions are meant for all healthcare professional participants, which are indicated at the end of the document by section.

General

**Formalities**

Introductions of interviewers

Express gratitude to the participant for taking the time to participate

Ask if the participant had enough time to read the study information and if they have any questions regarding it

Signing of informed consent form

**Introduction**

1) Tell us a little about yourself, what is your background? *Example education and experience* a) What is your role and title here? How long have you worked in this position?

b) Could you share with us a typical day for you?

2) What does your patient population look like? a) How do you classify them to be able to effectively manage them? *Example GOLD standards*

3) Do you use the ABCD assessment tool? If no what severity classification system do you use? Skip to question 3. i) Do you think this is a useful guideline?

ii) How do use the ABCD tool in your everyday practices?

**Basic Practices and Processes**

1) How is the patient referred to you? a) How do you access patient data?

b) How do you follow-up with your patients? i) In which situations?

ii) At which frequency?

2) How are you notified that your patient is having an event? a) What is your process for responding?

3) What outpatient services are you providing to your patients to help them manage their condition at home? a) In general, what else could be done for your COPD patients when they are at home to increase their Quality of Life?

*b)* Do you have some specific services or actions in mind? *Example smoking cessation*

4) What patient education do you perform? a) At what point during patient management do you educate your patients? And in what setting? *Example first visit, follow-up, after exacerbation*

b) What topics are you educating your patients on? Do you feel there is a topic that is much more important than the others?

c) How much effort goes into educating a patient?

a) Stable patients?

b) Deteriorating patients?

a) Who is responsible for palliative care?

How often do you perform spirometry in the hospital for:

Where is palliative care performed? *Example Nursing home, hospice*

**Clinical Strategies for Guideline Implementation and Practice**

1) Are smoking cessation strategies implemented in your work practices? Why or why not? a) If smoking cessation is not part of clinical practices skip to question 2. If smoking cessation is part of clinical practices and was overlooked on the survey, continue below.

b) Are you familiar with the 5A Guidelines of smoking cessation? i) How do you use the 5A’s tool in your everyday practices?

c) How do you advise on smoking cessation for the different patient severity groups? Do you ever suggest any tools? If so, which ones. If not, why not?

d) How many minutes do you speak with your patient to provide smoking counseling? Do you think this should be part of self-management or a separate process?

e) What sort of support from informal caregivers do you look for when providing smoking cessation? *Example Supportive family members*

2) Do you track patient medication adherence? If no, why not? If yes, how? a) What could help to support you in your effort to ensure that patients are adherent with your medication advice?

b) What measures do you take to prevent drug interactions?

c) Do you teach inhaler techniques? i) If yes, please describe the techniques. Who educates the patients on these techniques?

ii) If not, do you see it as an opportunity for better practice?

3) In general, how many of your patients have comorbidities? What type of comorbidities? a) How do you manage these comorbidities in your clinical practices?

b) Do you work with any other professionals to manage comorbidities? How?

c) How do you think the management of your patient and their comorbidities would change if you were able to remotely monitor your patient?

4) Do you have any physical activity programs for patients? If so, please describe them. If not, why not? a) How do you advise on physical activity for the different patient severity groups?

How do you decide if a patient should be referred to pulmonary rehabilitation or self-management? What criteria must they meet?

**Patient Management and Telehealth**

1) How do you think the management of your patient would change if you were able to remotely monitor your patient? a) Would it help you? In what way?

2) What sort of data would be useful for your job to be sent to you remotely? *Example Symptoms or self-recorded data.* a) How frequently would you need access to patient data to support your work practices? *Example Daily, on-going*

b) How do you feel about monitoring a patient remotely?

c) What do you think about the patient’s experience being monitored remotely? *Example their level of satisfaction, how it impacts their health etc.*

3) Have you ever suggested a patient to use an intervention for self-management or education? If so what tools? If not, why not? a) How do patients respond to these tools? *Example do you see improvements over time, do they seem uninterested*

b) How important are patient-reported outcomes to you?

c) Do you base any of your decisions on patient-reported outcomes? If yes, what decisions? If not, why not?

4) What do you think telehealth is? Please include any examples that might come to mind.

Participant Specific Questions

This section is for specific healthcare professional participants as noted by the headings.

**Nurse/Pulmonary Rehabilitation/Self-Management**

1) What decisions are you in charge of for patient management? *Example diagnostic or therapeutic* a) Do you help with patient education, self-management, or inhaler therapy? *Example a teaching related role*

**Pulmonary Rehabilitation/Self-Management**

2) What components of pulmonary rehabilitation/self-management do you combine in your practice? *Example physical activity, behavior change etc.* a) Do you think telehealth could be a useful component for you? i) If you see it as useful, what would you need to do to make it regular practice?

**Pulmonologist**

1) What, if any, self-management tools do you see as suitable for integrating into your everyday practices? *Example smoking cessation* a) How would you assign tasks of supporting self-management tools and to whom?

2) After you discharge the patients from the hospital, is there a procedure for following up with the patient? If yes, what is it? If no, why not? a) How do you schedule a follow-up with a patient? Who initiates first contact?

b) Does the frequency of scheduling change based on the patient’s status?
